# Tertiary Origin and Pleistocene Diversification of Dragon Blood Tree (*Dracaena cambodiana*-Asparagaceae) Populations in the Asian Tropical Forests

**DOI:** 10.1371/journal.pone.0060102

**Published:** 2013-04-01

**Authors:** Jian-Li Zhao, Lu Zhang, Selvadurai Dayanandan, Shivaprakash Nagaraju, Dong-Mei Liu, Qiao-Ming Li

**Affiliations:** 1 Laboratory of Plant Phylogenetics and Conservation, Xishuangbanna Tropical Botanical Garden, The Chinese Academy of Sciences, Kunming, Yunnan, People’s Republic of China; 2 Key Laboratory of Tropical Forest Ecology, Xishuangbanna Tropical Botanical Garden, The Chinese Academy of Sciences, Menglun, Yunnan, People’s Republic of China; 3 The Graduate University of The Chinese Academy of Sciences, Beijing, People’s Republic of China; 4 Department of Biology, Concordia University, Montreal, Quebec, Canada; CNR, Italy

## Abstract

**Background:**

The origin of extraordinarily rich biodiversity in tropical forests is often attributed to evolution under stable climatic conditions over a long period or to climatic fluctuations during the recent Quaternary period. Here, we test these two hypotheses using *Dracaena cambodiana,* a plant species distributed in paleotropical forests.

**Methods:**

We analyzed nucleotide sequence data of two chloroplast DNA (cpDNA: *atp*B*-rbc*L and *trn*D*-trn*T) regions and genotype data of six nuclear microsatellites from 15 populations (140 and 363 individuals, respectively) distributed in Indochina Peninsular and Hainan Island to infer the patterns of genetic diversity and phylogeographic structure. The population bottleneck and genetic drift were estimated based upon nuclear microsatellites data using the software programs BOTTLENECK and 2MOD. The lineage divergence times and past population dynamics based on cpDNA data were estimated using coalescent-based isolation-with-migration (IMa) and BEAST software programs.

**Results:**

A significant phylogeographic structure (*N*_ST_ = 0.876, *G*_ST_ = 0.796, *F*_ST-SSR = _0.329, *R*_ST = _0.449; *N*_ST_>*G*_ST_, *R*_ST_>*F*_ST-SSR_, *P*<0.05) and genetic differentiation among populations were detected. Bottleneck analyses and Bayesian skyline plot suggested recent population reduction. The cpDNA haplotype network revealed the ancestral populations from the southern Indochina region expanded to northward. The most recent ancestor divergence time of *D. cambodiana* dated back to the Tertiary era and rapid diversification of terminal lineages corresponded to the Quaternary period.

**Conclusions:**

The results indicated that the present distribution of genetic diversity in *D. cambodiana* was an outcome of Tertiary dispersal and rapid divergence during the Quaternary period under limited gene flow influenced by the uplift of Himalayan-Tibetan Plateau and Quaternary climatic fluctuations respectively. Evolutionary processes, such as extinction-recolonization during the Pleistocene may have contributed to the fast diversification in *D. cambodiana*.

## Introduction

Understanding the origin of extraordinary biological diversity in tropical forests remains as one of the greatest challenges in evolutionary biology. In general, two contrasting hypotheses have been put forward to explain the origin of high biological diversity in tropical ecosystems. The persistence hypothesis stipulates that the gradual evolution during the long-term survival of organisms and low extinction rates under stable climatic conditions may have lead to the present day high biological diversity in the tropics. Alternatively, the refugia hypothesis suggests that the current diversity in the tropics could be attributable to successive isolation and subsequent expansion of populations in response to frequent oscillations of the climate during the Quaternary period [1], [2], [3], [4], [5], [6]. Thus, the persistence hypothesis suggests that the origin of high biodiversity in the tropics should date back beyond the Quaternary period, whereas the refugia hypothesis stipulates that the biological diversification in the tropics is more recent and coincides with the Quaternary climatic fluctuations. Numerous studies suggest that the high biological diversity in the tropics may have originated during the pre-Quaternary era [1], [2], [4], [6], [7]. However, several examples, particularly from the Neotropics, support the refugia hypothesis suggesting a recent diversification during the Quaternary period [1], [2], [7], [8]. In contrast to the Neotropics, the evolution of biological diversity in the Asian Paleotropics may have been influenced by major geological events including the collision of the Deccan plate with Eurasia and associated topographical alterations during the Tertiary [6], [9], [10], [11], [12], [13]. Our present understanding of the post-glacial time evolution of plants and animals is mostly based on studies focusing on temperate and sub-tropical organisms, and limited data are available on the post-glacial evolution in paleotropical ecosystems.

The limited understanding of the evolution of tropical plants during the Quaternary period could be attributable to paucity of reliable data from the tropical regions. The fossil records of vascular plants in the tropics representing the Quaternary period are rare [14] and pollen data may not accurately identify the responses of plants to changes in the climate during the Quaternary period [15], [16]. As a means of overcoming these limitations, DNA based molecular genetic approaches have become an integral tool in understanding the genetic diversification and historical plant demographics. DNA- based genomic data of extant species are being used to infer genetic diversification and biogeographical history over the past several million years and provide a basis for inferring historical population demographics [17]. In particular, molecular dating based on chloroplast DNA (cpDNA) sequences and nuclear microsatellites provides a unique opportunity to explore the historical demography and chronology of evolutionary changes [15], [16], [18], [19], [20]. In Angiosperms, cpDNA is generally maternally inherited and dispersed through seeds, and therefore cpDNA markers provide a means to study seed-mediated species migration patterns [14], [21]. The biparentally nuclear microsatellite (nSSR) data provide a means to investigate fine-scale genetic structure attributable to the combined effects of both seed and pollen flow.

*Dracaena cambodiana* Pierre ex Gagnepain (Asperagaceae), commonly known as the dragon blood tree is a vulnerable species and generally distributed in the escarpments of island-like limestone mountains in paleotropical Southeastern Asia. Current dragon trees mainly distributed in Asia and Africa [22], [23]. *D. cambodiana* was the representative of dragon trees in Asia. Dispersal of tropical elements between Asia and Africa has two possible ways [6]. One was megathermal rainforest expansion during warm Paleocene and Miocene. The other one was Indian plate collision to Eurasian land during Miocene. Whatever the ways for *D. cambodiana*, this species should exist in Asian Paleotropics during Tertiary. Thus, *D. cambodiana* serves as an ideal model species to test the biodiversity-original hypotheses of plant species in Paleotropics. The objectives of this study are (i) to illustrate the genetic diversity, gene flow and past population demographics using cpDNA and nSSR; (ii) to determine the ancestral populations and past migration patterns through phylogeographic analysis of cpDNA; (iii) to date the genetic divergence under different molecular clock methods.

## Materials and Methods

### Ethics Statement

*Dracaena cambodiana* is national second class protected plants in China. This species is listed in the Inventory of Rare and Endangered Plants of China and the Key Protected Inventory of Wild Plants of China (http://db.kib.ac.cn/eflora/View/plant/ZXBWSpecies.aspx), but it is not evaluated by the International Union for Conservation of Nature (IUCN) and it is also not recognized as the endangered or protected species in Thailand, Cambodia, Laos and Vietnam. Furthermore, the locations in Thailand, Cambodia, Laos and Vietnam are not privately-owned or protected in any way except the Pumat National Park of Vietnam. We got the permission of the Wildlife Protection and Administration Office under the Forestry Department of Yunnan, Guangxi and Hainan Province in China and the permission of the Administration of Pumat National Park of Vietnam. We also got the permission of local forestry department in other locations of Thailand, Cambodia, Laos and Vietnam. The sampling process was under the guidance of local rangers. This plant was solely used for scientific research and our sampling will not affect the regular growth of *D. cambodiana*.

### Sample Collection and DNA Extraction

**Figure 1:**
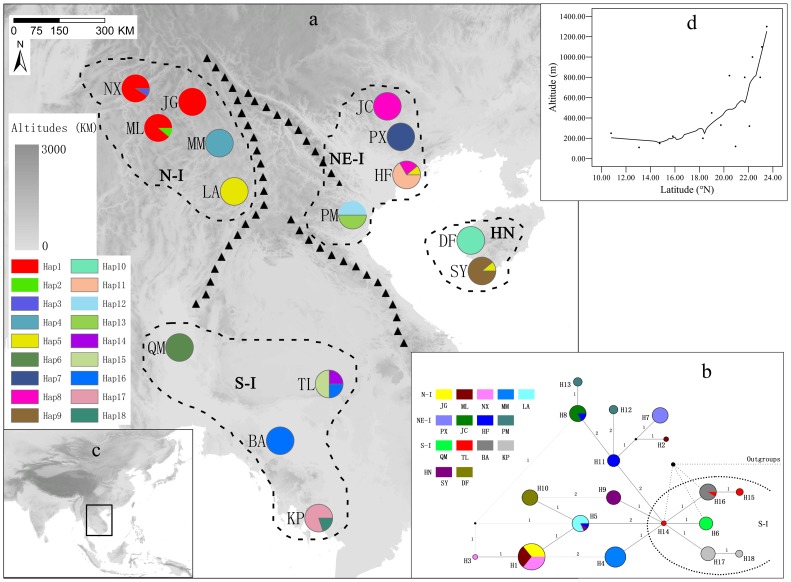
Geographic distribution and evolutionary relationships of 18 cpDNA haplotypes in *Dracaena cambodiana*. **a:** Different colors in pies charts represent haplotypes. The dashed lines indicate the division of fifteen populations into four groups based upon their geographic location. Black triangles represent main geographical barriers originated during the Tertiary era (modified from Lacassin *et al.,* 1993; Huchon *et al.,*1994; Searle, 2006). **b:** Colorsrepresent populations. The black dots are the missing haplotypes or missing samples. The numbers on the branches represent number of mutations between two connected haplotypes. The gray connected lines among haplotypes were resolved based on the methods of Crandall & Templeton (1993). Segmented-elliptic-dashed lines represent the cluster of S-I populations. Straight-dashed line is the connection to outgroups. The detailed connections of outgroups is given in Figure S2. **c:** general position of map A in Southeast Asia. **d:** The trend of altitudes shifting along latitudes.

Leaf samples of *D. cambodiana* plants from 15 localities representing four geographical regions covering most of the natural distribution range of the species in Asia (Figure 1a, Figure 1d, Table 1) were collected, and dried using silica gel. The total genomic DNA was extracted from about 100 mg of silica-gel-dried leaf materials following a CTAB based DNA extraction protocol [24].

**Table 1:** Sampling information of *Dracaena cambodiana* for cpDNA and nuclear microsatellite (nSSR) analyses.

Code	Population	Latitude (N)/Longitude (E)	Altitude (m)	N cpDNA/nSSR	cpDNAhaplotypes (no. of individuals)	
**S-I Southern Indochina**				**31/84**		
QM	Chiang Mai, Thailand	15°50′/100°12′	220	7/−	H6(7)	
TL	Ubon, Thailand	14°45/104°38′	150	4/28	H14(1), H15(2), ***H16(1)***	
BA	Batdambang, Cambodia	13°04′/103°11′	110	10/28	***H16(10)***	
KP	Kampot, Cambodia	10°47′/104°19′	250	10/28	H17(8), H18(2)	
**NE-I Northeastern Indochina**				**34/78**		
PX	Pingxiang, Guangxi Province, China	22°05′/106°45′	320	10/24	H7(10)	
JC	Jingxi, Guangxi Province, China	22°58′/106°21′	800	9/24	***H8(9)***	
HF	Haiphong, Vietnam	20°57′/106°57′	120	9/30	***H5(1)***, ***H8(2)***, H11(6)	
PM	Pumat National Park, Vietnam	19°45′/105°19′	330	6/−	H12(3), H13(3)	
**N-I Northern Indochina**				**56/156**		
JG	Jinggu, Yunnan Province, China	23°06′/100°35′	1100	10/23	***H1(10)***	
ML	Menglian, Yunnan Province, China	22°20′/99°34′	1000	9/42	***H1(8)***, H2(1)	
NX	Zhenkang, Yunnan Province, China	23°30′/98°54′	1300	11/30	***H1(10)***, H3(1)	
MM	Mengyuan, Yunnan Province, China	21°42′/101°22′	800	17/30	H4(17)	
LA	LouangPhrabang,Laos	20°27′/101°49′	817	9/31	***H5(9)***	
**HN Hainan Island**				**19/45**		
DF	Dongfang, Hainan Province, China	19°00′/108°49′	450	10/24	H10(10)	
SY	Sanya, Hainan Province, China	18°17′/109°09′	200	9/21	***H5(1)***, H9(8)	
Overall		–	–	**140/363**	H1-18 (140)	

The shared haplotypes are indicated in ***bold-italic***.

(−) = no individuals were available for nuclear microsatellite analysis.

### Molecular Markers

#### Chloroplast DNA (cpDNA) sequences

Ten individuals representing different geographic regions were surveyed for the nucleotide sequence variation at four cpDNA regions (*trn*L*-trn*F, *psb*A*-trn*H, *atp*B*-rbc*L, and *trn*D*-trn*T). Based upon sequence variability, *atp*B*-rbc*L [25] and *trn*D*-trn*T [26] regions were chosen for further analysis. These regions were PCR amplified, purified and sequenced (see online supplement for details). The DNA sequences were aligned using CLUSTALW [27] as implemented in MEGA V5 [28] and manually edited to improve the alignment. Insertions and deletions (indels) in the repeated regions were excluded from the analyses as these regions are known to evolve rapidly and prone to homoplasy 29,30. A total of 140 individuals were sequenced and representative cpDNA haplotype sequences of *D. cambodiana* and outgroups were deposited in the GenBank (accessions: JF784389–JF784419).

#### Nuclear microsatellite genotyping

Six polymorphic nuclear microsatellite loci, *DC003*, *DC006*, *DC140*, D*C437*, *DC460*, *DC501* were used for genotyping a total of 363 individuals following the protocol of [31].

### Genetic Analyses

#### Diversity parameters

The sampled *D. cambodiana* populations were clustered into four groups based upon their geographical distribution (Figure 1).

For cpDNA data, the haplotype diversity (*h*) and nucleotide diversity (*π*) were calculated for each group and overall level using the program DNASP V5.10 [32]. The total genetic diversity (*H*_T_) and within population genetic diversity (*H*_S_) were calculated using PERMUTCPSSR V2.0 [33]. The genetic differentiation (*F*_ST-cp_) among all fifteen populations and among four groups was calculated using ARLEQUIN V3.1 [34].

For microsatellite data, the presence of null alleles, large allele dropout and scoring errors due to stuttering were tested using MICRO-CHECKER V2.2.3 [35] and anomalies were rectified following Brookfield [36]. The heterozygosity (observed *H*_O_ and expected *H*_E_) and test for linkage-disequilibrium were calculated using GENEPOP V3.3 [37]. The deviations from Hardy-Weinberg equilibrium values were tested using FSTAT V2.9.3.2 [38] by calculating *F*_IS_ values for each population and each locus. The statistical significance of deviations was assessed by randomization for 1000 times. The proportion of genetic variation partitioned among populations and among groups of populations were quantified using analyses of molecular variance (AMOVA) as implemented in ARLEQUIN and the statistical significance was tested with 10000 permutations.

#### Population differentiation and phylogeographic structure

The presence of phylogeographic structure of cpDNA was tested by comparing the level of population differentiation based upon the infinite allele model (*G*_ST_) with an estimate of population subdivision based on phylogenetically ordered alleles (*N*_ST_) using the program PERMUTCPSSR. Because *G*_ST_ may be problematic for the interpretation of genetic differentiation, we employ the standardized genetic differentiation (*G^'^*_ST_) to measure genetic differentiation among populations according to the formula *G^'^*_ST_ = *G*_ST_(1+ *H*_S_)/(1 - *H*_S_) [39].

Using nuclear microsatellite data, we assessed the genetic structure based on two differentiation indices namely *F*_ST-SSR_ [40] and *R*_ST_ [41]. The *F*_ST-SSR_ value is based on infinite allele model and *R*_ST_ estimate is based on the stepwise mutation model. The significant difference between these two estimates is an indication of the existence of phylogeographic structure. The genetic structure was inferred using SPAGEDI software [42]. We tested for the presence of isolation-by-distance (IBD) by regression analysis between genetic differentiation *F*_ST_/(1-*F*_ST_) and the natural logarithm of geographic distance for all pairs of populations [43]. The statistical significance of IBD was tested using the Mantel test with 1000 permutations as implemented in NTSYS V2.10e [44]. We tested the phylogeographic signal using nuclear microsatellite data following Hardy *et al.* [45] as implemented in SPAGEDI. This approach is based on comparison between *F*_ST-SSR_ and *R*_ST_ which are expected to be equal under the null hypothesis of no phylogeographic signal. *G^'^*_ST_ of nSSR was estimated by SMOGD [46].

#### Detection of genetic clusters

We used nuclear microsatellite data to test for the existence of cryptic population structure employing the Bayesian model based software program STRUCTURE V2.3.3 [47]. An admixture model with correlated allele frequencies was used for estimating the historical population admixture and number of genetic clusters (*k*) ranging from 1 to 20 [48], [49]. All analyses employing the STRUCTURE program were performed with 20 replicates, each with a burnin period of 20 000 and a Markov chain Monte Carlo (MCMC) value set at 130000. These values were large enough to stabilize log (alpha), Ln likelihood and the MCMC chain converging to consistent end results [47]. The method of Evanno *et al*. [50] was used for finding the most likely value of *k* by plotting the log probability of L(*k*) and the Δ*k* of the data over multiple runs using the program STRUCTURE HARVESTER [51]. In order to compare runs with the same value of *k*, we calculated the symmetric similarity coefficients (SSC) using the Greedy algorithm as implemented in the CLUMPP software [52]. The groups of runs with SSC ≥0.8 were combined and bar plots were prepared using the software program DISTRUCT [53]. The GenAlex program [54] was used for calculating pairwise genetic distances between individuals [55] and principal coordinate analysis (PCA).

#### Haplotype network analyses

The evolutionary network reconstruction and ancestral haplotype inference based on cpDNA data were carried out using the software program NETWORK V4.5.1.6 (www.fluxus-engineering.com) following the median-Joining method [56]. The ambiguous loops were resolved following the method of Crandall & Templeton [57] assuming that rare haplotypes and widespread haplotypes are more likely located at the tip and interior respectively and singletons are more likely derived from the same population. We chose *Agava* sp., *Yucca gloriosa* and *Asparagus setacea* as outgroups for the evolutionary network analysis. Phylogenetically *Asparagus* is basal to *Agava* and *Yucca*, which are closely related to *Dracaena* [58]. Thus we used *Asparagus* as the basal outgroup to root the evolutionary network.

#### Molecular dating and demographics of cpDNA

We used the ‘Isolation with migration’ (IM) coalescent model as implemented in the program IMa2 [59], [60] to estimate the divergence times of four geographically distinct groups of populations. We calculated the divergence time, *t*_i_ (i as the node of diverging groups) and the time since most recent common ancestor (TMRCA) of the species and each group (*t*_TMRCA_). The resulting values were converted to absolute time scale, *T* in years using the formula *T* = *t*/*µk*, where *μ* is the number of substitution per site per year (s/s/y) and *k* is the length of the sequence [61]. We estimated *μ* as 0.146476±0.063280×10^−9^ s/s/y based on uncorrelated exponential clock model (Table S1 and File S1), and the value of *k* is 1814 bp. The newick format of a tree for IMa2 based on pairwise *F*_ST_ values among four groups (Table S2) was constructed using NTYSYS software package. The haplotype in Southern Indochina (S-I), which was found to be ancestral based upon haplotype network analysis was used for rooting the haplotype network.

The departure from population demographic equilibrium was assessed using Tajima's *D* [62] and Fu's *F_S_* [63] statistics using the ARLEQUIN software. The past population dynamics and the estimates of most recent common ancestor or *T*_TMRCA_ were further analyzed through Bayesian skyline plot [64] as implemented in the program BEAST V1.5.4 [65]. The Bayesian skyline reconstruction and lineages through time analysis were conducted using TRACER V1.5 [66] with burn-in of 10% of chains. The details of IMa2 and BEAST analysis procedure are given in File S1. The evidence for population demographic growth was investigated through mismatch distribution analysis using the software program ARLEQUIN. The pair-wise mismatch distribution is expected to be multimodal in samples drawn from populations at demographic equilibrium and unimodal in populations that have undergone a recent demographic expansion [67], [68] or a range expansion with high levels of migration between neighboring populations [69]. The goodness-of-fit of mismatch distributions to the expected distribution under a sudden expansion model [68] was tested using the sum of squared deviations (SSD) and the raggedness index [HRag; 70].

#### Population bottleneck analyses of nSSR

The excess in heterozygosity (*H*_e_) as compared to the expected heterozygosity under mutation drift equilibrium (*H*_eq_) for a given allelic diversity is an indication of recent reduction in the effective population size [71], [72]. We used the software program BOTTLENECK [73] to assess any significant excess in heterozygosity (*H*_e_>*H*_eq_) through Wilcoxon sign-rank test and sign test with 5000 replications. We performed the analysis under Infinite allele model (IAM) and two-phase model of microsatellite evolution with the proportion of single step stepwise mutation (SMM) set to 70% and the variance to 30%. The population bottleneck is also expected to change the allele frequency distribution [74]. The allele frequency mode-shift indicator test was performed to test for significant departure in allele frequency distribution as compared to equilibrium expectations.

The population divergence under genetic drift alone or the balance between gene flow and genetic drift was assessed using the software program 2MOD V0.2 [http://www.rubic.rdg.ac.uk/~mab/software.html]. The genetic drift model assumes that fragmented populations are diverging by drift alone, and the gene flow–drift model assumes population allele frequencies are determined by the balance between genetic drift and gene flow. Two independent runs of 500000 iterations were performed and the initial 10% of the iterations were used as the burn-in. The Bayesian factors were calculated using TRACER to test the relative contribution of drift and gene flow to the population divergence.

## Results

### Genetic Diversity

The length of aligned consensus sequences of cpDNA fragments, *atp*B-*rbc*L and *trn*D-*trn*T of 140 individuals representing 15 *D. cambodiana* populations were 858 bp and 990 bp respectively. The *atp*B-*rbc*L region included two 1bp indels and the *trn*D-*trn*T region included one 8 bp (only in one individual of the population TL), one 17 bp, one 5 bp and two 1bp indels. After excluding gaps, the length of combined cpDNA consensus sequence length was 1814 bp, which included 15 polymorphic sites and 18 haplotypes (Table 1; Figure 1).

Overall, only 4 (H1, H5 H8, H16) of the 18 haplotypes were shared among populations (Table 1; Figure 1). Only one haplotype (H5) was shared among three populations (HF, LA, SY) in different groups (NE-I, N-I, HN). The other three haplotypes were shared between two or three populations within groups (H1: NX, ML, JG; H8: JC, HF; H16: TL, BA). The remaining 14 haplotypes were fixed within each population. The haplotype diversity and nucleotide diversity of cpDNA were 0.911 and 1.81×10^−3^ respectively (Table 2). The total genetic diversity was 0.968. The genetic diversity within population was 0.198. Observed heterozygosity (*H*_O_) estimated from nSSR was from 0.474 to 0.706 (average was 0.637). Expected heterozygosity (*H*_E_) estimated from nSSR was from 0.747 to 0.919 (average was 0.948).

**Table 2:** Genetic diversity and phylogeographic structure estimated from cpDNA and nSSR data of *Dracaena cambodiana*.

Group	cpDNA						nSSR				
	*H*_S_	*H*_T_	*h*	*π*×10^−3^	*G*_ST_	*N*_ST_	*H*_O_	*H*_E_	*F*_ST-SSR_	*R*_ST_	*F*_IS_
S-I	0.297	0.958	0.772	0.86	0.690	0.807	0.580	0.856	0.343	0.321	0.080
NE-I	0.289	0.963	0.784	1.35	0.700	0.732	0.706	0.890	0.298	0.438	0.011
N-I	0.081	0.739	0.643	0.72	0.891	0.817	0.678	0.919	0.324	0.406	0.047
HN	–	–	0.573	0.63	–	–	0.474	0.747	0.395	0.701	0.170
Overall	0.198	0.968	0.911	1.81	0.796	0.876*	0.637	0.948	0.329	0.449*	0.050

Genetic diversity for each loci and each population was summarize in Table S3 and Table S4.

*H*_S_, genetic diversity within populations; *H*_O_, observed heterozygosity; *H*_E_, expected heterozygosity; *H*_T_, total genetic diversity; *h*, haplotype diversity; *π*, nucleotide diversity; *G*_ST_, *N*_ST_, *F*_ST-SSR_ and *R*_ST_, genetic differentiation for and phylogeographic signal test; *, *N*_ST_>*G*_ST_, *F*_ST-SSR_>*R*_ST_; *P*<0.05, means significant phylogeographic structure; *F*_IS_, inbreeding coefficient; -, Populations that are smaller than two cannot be analyzed in the PERMUTCPSSR program.

### Population Differentiation and Phylogeographic Structure

The hierarchical AMOVA based upon both cpDNA and microsatellites data (Table 2 and Table 3) revealed significant species level genetic structure (*F*_ST-cp_ = 0.850, *P*<0.05; *F*_ST-SSR = _0.329, *P*<0.01) and within–group population divergence (*F*_SC-cp_ = 0.833, *P*<0.01; *F*_SC-SSR = _0.322, *P*<0.01). Although cpDNA data revealed a significant genetic differentiation among four groups (*F*_CT-cp_ = 0.104, *P*<0.01), microsatellite data showed no significant genetic differentiation among groups (*F*_CT-SSR_ = 0.001, *P*>0.05). The gene flow (*N*m) among populations based on cpDNA and microsatellite data was 0.0882 and 0.5099 respectively. The range-wide isolation by distance (IBD) in *D. cambodiana* was statistically non-significant (r_cp_ = 0.027 *P* = 0.437; r_SSR_ = 0.199 *P* = 0.084). A significant phylogeographic structure based on both cpDNA and microsatellites data was detected (*G*_ST-cp_ = 0.796, *N*_ST-cp_ = 0.876, *N*_ST-cp_>*G*_ST-cp_, *P*<0.05; *F*_ST-SSR = _0.329, *R*_ST-SSR = _0.449, *R*_ST-SSR_>*F*_ST-SSR_, *P*<0.05; Table 2). *G^'^*_ST_ of cpDNA was approached to 1 and *G^'^*_ST_ of SSR was 0.921.

**Table 3:** Results of Analysis of Molecular Variance (AMOVA, cpDNA/nSSR) of *Dracaena cambodiana*.

Source of variation	d.f.	Sum of squares	Variance components	Percentage of variation	Fixation Indices
Among groups	3/3	15.94/125.78	0.05/0.002	10.39/0.10	*F*_CT_ = 0.104*/*F*_CT = _0.001
Among populations within groups	11/9	38.10/392.27	0.37/0.75	74.62/32.22	*F*_SC_ = 0.833*/*F*_SC = _0.322*
Within populations	125/350	9.26/549.79	0.07/0.01	15.00/0.22	*F*_ST-cp_ = 0.850*, *N*m = 0.0882/*F*_ST-SSR = _0.329*, *N*m = 0.5099#
Within individuals	−/363	−/566.50	−/1.56	−/67.46	

* *P*<0.05;

# for cpDNA, *N*m = 0.5 (1–*F*_ST_)/*F*_ST_, for microsatellite, *N*m = 0.25 (1–*F*_ST_)/*F*_ST_ (Wright 1931; Wright 1965).

### Genetic Clusters

**Figure 2:**
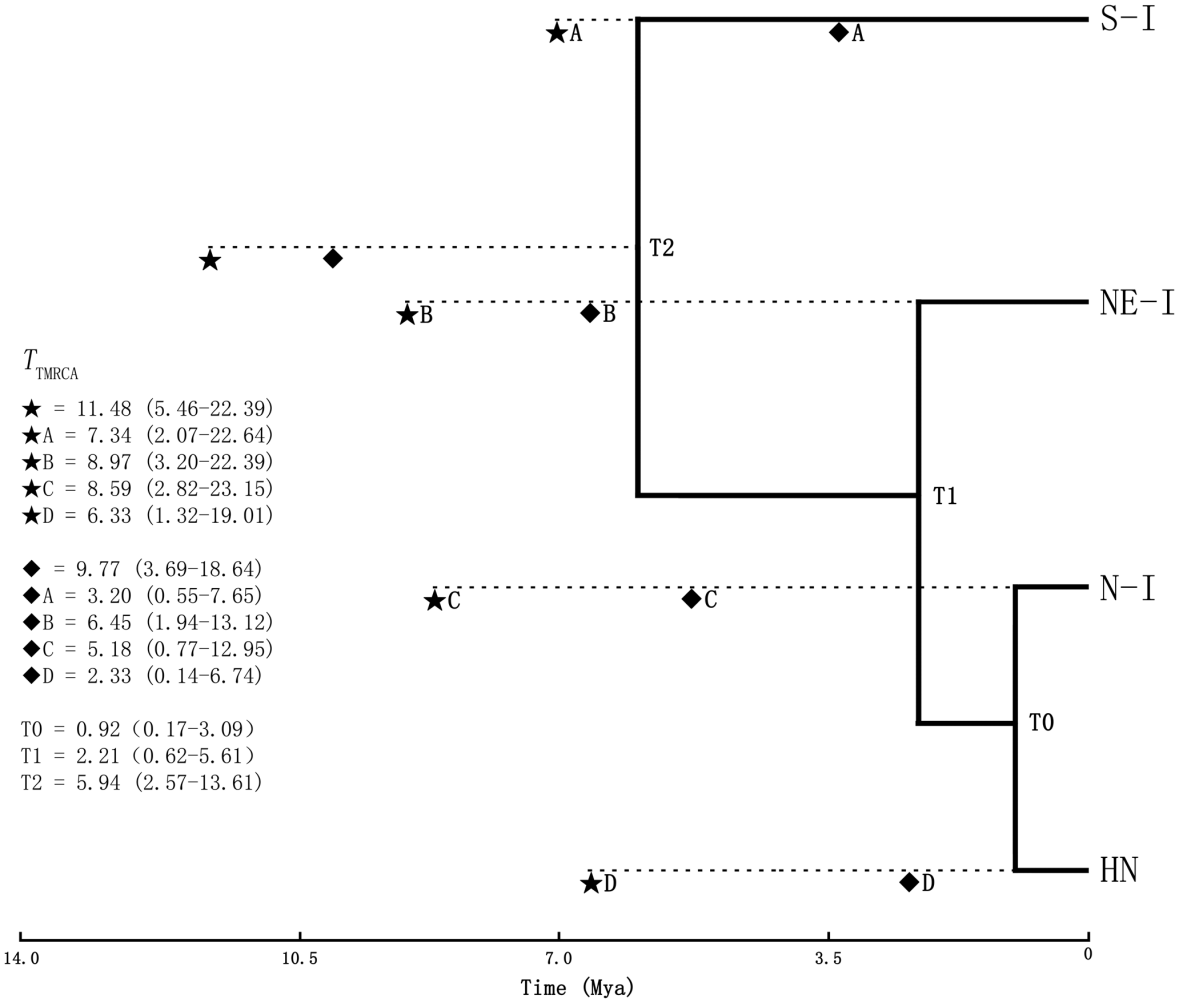
Results of Bayesian model based clustering of individuals using the STRUCTURE program. **a)** Distribution of likelihood value LnP(D) and the distribution of model parameter DeltaK (ΔK). All estimated values are based on 20 replicates and values of LnP(D)(±SD) and ΔK are plotted against its corresponding K. **b)** Barplots showing the results of the Bayesian cluster analysis. The colour in each barplot represents the probability of each individual to belong to an admixture group. Populations are ordered based on their population IDs as given in Table1.

In the STRUCTURE analysis, the posterior probability (LnP(D)) gradually increased reaching the highest value corresponding to K = 9 (Figure 2A). Similarly, the ΔK value also showed the highest peak at K = 9 (Figure 2A). Although few peaks of LnP(D) at K values of 12, 15 and18 were present, we chose K = 9 as the optimal number of genetic clusters. The value of K = 9 corresponded to the highest peak of LnP(D) at the lowest K value and the highest ΔK value, the criteria for choosing the optimal K value as recommended in the documentation of STRCTURE software (http://pritch.bsd.uchicago.edu/software). The occurrence of nine genetic clusters (K = 9), a value higher than the geographically defined four sampling regions suggests a high genetic differentiation among populations than among regions. The bar-plot depicting ancestry of samples (Figure 2B) further indicated that the populations from Northeastern Indochina were more or less genetically similar to Northern Indochina and represents genetically diverse group. The populations in the Hainan Island and Southern Indochina showed complex genetic admixture with membership in more than one cluster. The population in the Hainan Island represents a genetically distinct group as compared to other populations analyzed in the present study.

### Haplotype Network Analysis

The rooted phylogenetic network analysis of 18 cpDNA haplotypes (Figure 1b and Figure S2) showed that only haplotypes from the S-I group connected to the outgroups. The haplotype H14 showed most connections with other haplotypes suggesting haplotypes in S-I are derived from haplotype H14. This suggests that the S-I group is ancestral [75]. The haplotype networks of other three groups are complex and formed several loops. The connections among H3, H5, and H8 were resolved following the methods of [57]. With the exception of S-I group, the phylogenetic clustering of other three groups was not congruent with their geographic locations. The relationships among haplotypes H1, H4, H9 and H10 were clearly interpretable based on their geographic location after resolving the loops following the minimum evolution theory. Most haplotypes in the Northeastern group (NE-I) have derived from H11 and one haplotype, (H5) has derived from H14. Haplotypes in the Northern Indochina group (N-I) have derived from three ancestral haplotypes: H1 from H5, H2 from H11and H4 from H14.

### Molecular Dating and Historical Demography

**Figure 3:**
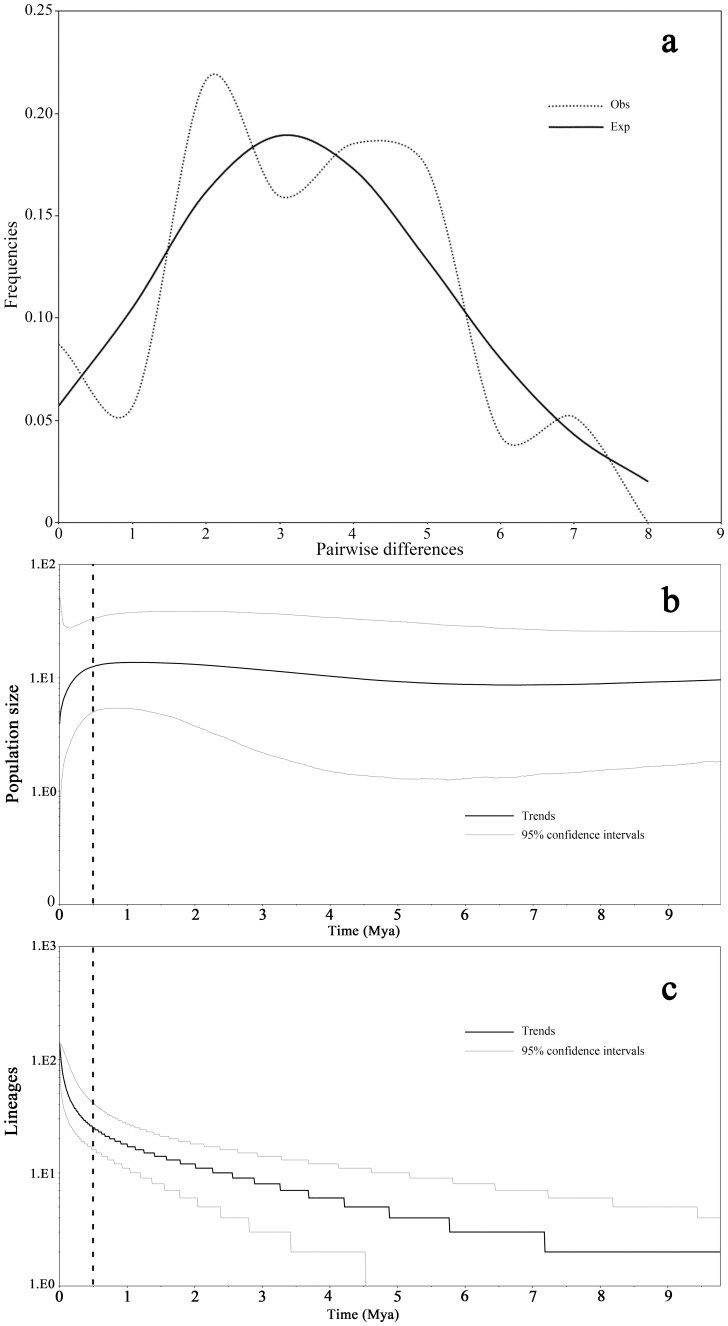
The Phylogenetic tree representing evolutionary relationships of four geographically based groups of *Dracaena cambodiana* populations. The coalescent times of the most recent common ancestor (*T*_TMRCA_) for overall populations and each group (A, B, C, D) based on IMa2 (★)and BEAST (♦) analyses. The divergence times among the four geographic groups are T0 T1 and T2. Dashed lines corresponds to *T*_TMRCA_. The 95% highest posterior density intervals (95%HPD) are given in parentheses. The unit time is in million years ago (Ma).

**Figure 4:**
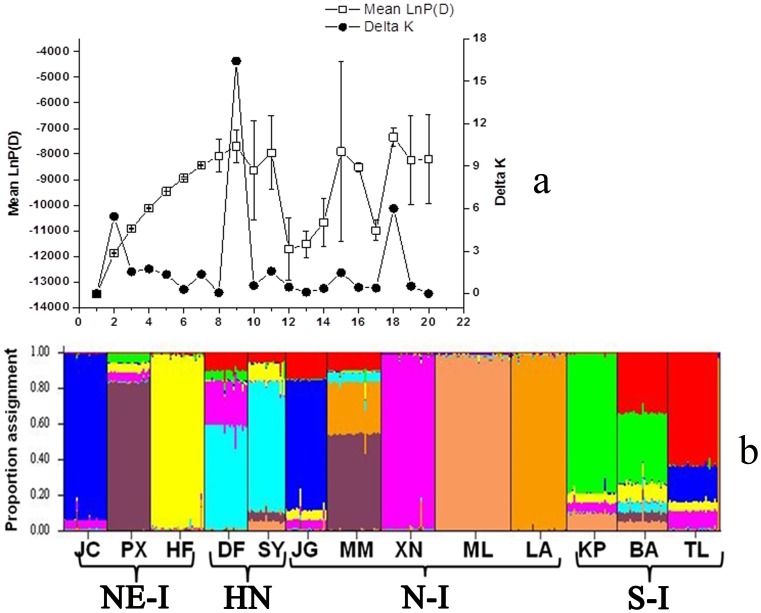
Population demographics of *Dracaena cambodiana*. **a:** mismatch distribution. dashed lines are observed and black lines are expected values. **b** (change in population size over time) and **c** (divergence of lineages): x-axis is the median time in millions years ago (Ma); black lines represent median estimate and gray lines represent 95% confidence intervals.

The coalescent times of the most recent common ancestor (*T*_TMRCA_) based on the results of IMa2 and BEAST program were 11.48 (5.46–22.39) million years ago (Ma) and 9.77 (3.69–18.64) Ma respectively (Figure 3). The *T*_TMRCA_ of the 18 cpDNA haplotyes was 15.80 (5.29–29.88) Ma. The posterior probability distributions of all parameters showed a high degree of convergence (Figure S1). These results suggest that the origin of *D. cambodiana* dates back to Tertiary period from Middle Oligocene to Miocene. Although coalescent times of four groups dates back to Tertiary, the terminal lineages show more recent divergence dating back to the Quaternary period (Figure 4c, Figure S3, and Figure S4).

The mismatch distribution was multimodal (Figure 4a), indicating a demographic equilibrium. The SSD value (0.011, *P*<0.05; Table 4) and HRag value (0.053, *P*<0.05) also supports the demographic equilibrium. Moreover, both Tajima’s *D* value (0.549, *P*>0.05) and Fu’s *F_S_* (−2.597, *P*>0.05) value also in agreement with the demographic equilibrium.

**Table 4:** The neutrality tests and mismatch distribution analysis within four groups and overall populations of *Dracaena cambodiana*.

Group	SSD	HRag	Tajima's *D*	Fu's *F_S_*
S-I	0.079	**0.263**	0.679	−1.169
NE-I	**0.074**	**0.264**	0.751	0.420
N-I	0.026	0.101	−0.003	−0.941
HN	0.132	0.483	0.875	1.473
Overall	**0.011**	**0.053**	0.549	−2.597

Tajima's *D* and Fu's *F_S_* and their significance values are reported. The sum of squared deviation (SSD) and raggedness index (HRag) are for testing the sudden expansion model. The bolds indicate *P*<0.05.

### Population Bottleneck Analysis

The population bottleneck analysis showed a mode-shift in allele frequency distribution in three populations (ML, MM and LA) from Northern Indochina (Table S5). These three populations (ML, MM and LA) along with two populations from Northeastern Indochina (PX and HF) and one population from Hainan Island (SY) showed significant deviation (Wilcoxon sign rank test, *P*<0.05; sign test, *P*<0.05) from mutation drift equilibrium (Table S5). Among these populations, two populations (ML and MM) showed strong evidence for recent population bottleneck with statistical significance in all three tests. The population divergence analysis using 2MOD software program highlighted genetic drift as a major evolutionary force in *D. cambodiana* (*P*_geneflow_ = 0.00014; Bayes Factor_geneticdrift vs. geneflow_ = 101.464).

## Discussion

### High Genetic Diversity and Limited Gene Flow

The genetic diversity estimates of *D. cambodiana* were higher than most high-altitude tropical, subtropical and temperate tree or shrub species in Southeast Asia [mean cpDNA HT = 0.79 in 76]. The diversity parameters (*H*_E_) and genetic differentiation (*F*_ST_) estimated using nuclear microsatellites were also high as compared to many tree species [77]. Plant species can survive during glacial periods in heterogeneous habitats [16] suggesting that the topologically heterogenous mountains may have served as reservoirs of genetic diversity as evident in *Lithocarpus* sp. in tropical Asia [78]. A high genetic diversity observed in *D. cambodiana* is in agreement with the postulation that high genetic diversity could be attributable to long-term evolution of plant populations isolation in refugia under fluctuating climatic conditions [14], [79], [80].

The limited seed dispersal and low levels of gene flow may act as a driving force of divergence among populations [81]. The rareness of shared haplotypes and prevalence of private alleles within populations of *D. cambodiana* indicate that the gene flow through seeds between populations is limited despite possessing berry type fruits adapted for bird-mediated long-distance dispersal. The flower of *D. cambodiana* is entomophilous and therefore pollen flow among distant populations is unlikely. The significant population genetic differentiation and pronounced larger *N*_ST_ than *G*_ST_ is an indication of limited gene flow among populations. High *G'*_ST_ of cpDNA and nSSR support the strong population structure with limited gene flow among populations.

Although our cpDNA haplotype data showed a high genetic divergence between populations in Hainan Island and Indochina Peninsular, the microsatellite data provided insight into contemporary gene flow. The significant differences between corresponding *F*_ST-SSR_ and *R*_ST-SSR_ values indicated that population subdivision between Hainan Island and Indochina Peninsular reflect a phylogeographic structuring. Similarly, *F*-statistics and Bayesian model based clustering also reveled strong differentiation between populations in Hainan Island and Indochina Peninsular (South, North and Northeast) corroborating the cpDNA-based results and providing evidence for limited gene flow between populations. The low *N*m value (<1) also suggests that the limited migration among populations may not offset the divergence due to genetic drift [82]. The bottleneck analysis supported the genetic drift biased genetic differentiation confirming the low levels of among-population gene flow. The significant positive *F*_IS_ for some of the microsatellite loci is an indication that inbreeding occurs in several populations [83]. Thus, strong genetic differentiation among *D. cambodiana* populations appears to be the result of the combined effect of distinctly limited gene flow and significant inbreeding within populations.

### Tertiary Origin and Rapid Divergence

Past geological events and associated climate change including the uplift of Himalayan-Tibetan Plateau and Quaternary glaciations [10], [11], [13] may have played an important role in the biological diversification, species extinction, speciation and distribution in the Asian tropics. The present topology of Asian mountains and the biogeographical boundaries in the Indochina Peninsula may have formed during the rapid uplift of Himalayan-Tibetan Plateau [11], [84], [85], [86], erecting barriers for plant migration [9], [12], [18]. The present geographical distribution of *D. cambodiana* is closely linked to the topographical features of the Indochina Peninsula (Figure 1a), indicating genetic divergence of *D. cambodiana* was significantly impacted by Tertiary extrusions of mountain. The mammalian fossils data of Tertiary origin also suggest that the middle Miocene environmental change and geological events related to the collision of the Deccan plate with Eurasia may have contributed to species extinction and speciation [87]. Although BEAST and IMa differ in the assumptions of genealogy samplers for coalescence process [88], these two coalescence-based methods yielded similar results of TMRCA (9.77–15.80 Ma), confirming the robustness of time estimates based on molecular dating. Such TMRCA dating suggests that regional genetic diversification of *D. cambodiana* are of Tertiary origin, correlated with the rapid uplift of the Himalayan-Tibetan Plateau at 7–10 Ma [11], suggesting the influence of the uplift of Himalayan-Tibetan Plateau on genetic divergence. The historical events along with relationship between latitude and altitude indicate that *D. cambodiana* may have migrated to the north from the south as a megathermal rainforest element before the geological uplift of the north (Figure 1d) tracking the temperature increase during the early Tertiary [6]. This suggest that the uplift of mountains may have contributed to the increase in genetic diversity in north Indochina Peninsula. These evidences suggest that the genetic stock resulting from initial differentiation of *D. cambodiana* during the Tertiary may have persisted and served as the source material for further diversification.

The Quaternary period (∼2.4 Ma to the present) is considered as one of the most important periods for genetic diversification and speciation [79], [80], [89] in a variety of ecosystems due to frequent changes in the climate and associated series of glaciations or ice ages [90], [91], [92]. The *T*_TMRCA_ of *Dysosma versipellis* haplotypes corresponds to Pleistocene era suggesting the effect of glacial and interglacial cycle on genetic divergence of plants [93]. Relatively rapid diversification of *D. cambodiana* terminal lineages with short branches corresponding to the Pleistocene period (Figure 4c, Figure S3 and Figure S4 ) indicates a rapid recent divergence of populations caused by Quaternary climate change [5], [17], [94]. However, skyline plot of cpDNA and bottleneck analyses of microsatellites suggested that *D. cambodiana* experienced a recent reduction in population, suggesting a Quaternary climate change also induced population size change. Thus, we inferred that evolutionary processes such as extinction-recolonization due to climate change during the Pleistocene may have contributed to the fast diversification in *D. combodiana*.

## Supporting Information

Figure S1 **The posterior probability convergence of** ***t*** **0,** ***t*** **1,** ***t*** **2 and** ***t*** **_TMRCA_.** A: convergence of time parameters for each node in Figure 3. B: convergence of time parameter for the most recent common ancestor.
(PDF)

Figure S2 **The phylogenetic network of haplotypes with outgroups.** O-1 = *Agava sp.*, O-2 = *Yucca gloriosa*, O-3 = *Asparagus plumosus*. Red dots are missing haplotypes or missing samples.
(TIF)

Figure S3 **The bayesian tree for evolutionary rate estimation based on uncorrelated lognormal relaxed clock.** Haps are the cpDNA haplotypes. The numbers above branches are the median rates of substitutions per site per million years. The numbers below the branches denotes the Bayesian posteriors. The height-median divergence time (boldfaces) and the 95%HPD time ranges (blue bars) marked at the nodes for the posteriors are more than 0.6. The coalescent times of the most recent common ancestor (*T*_TMRCA_) of 18 cpDNA haplotypes is 15.80 (5.29–29.88) Ma.
(TIF)

Figure S4 **The bayesian tree of** ***D. cambodiana*** **individuals based on Bayesian Skyline model.** The height-median divergence time (boldfaces) and the 95%HPD time ranges (blue bars) marked at the nodes. The coalescent times of the most recent common ancestor (*T*_TMRCA_) of all individuals is 9.77 (3.69–18.64) Ma.
(TIF)

Table S1 **log10 Bayes factors of three different relaxed clock models.**
(DOCX)

Table S2 **Pairwise genetic differentiation (** ***F*** **_ST_) among four groups.** The permutations for significance tests were 1023. *P*-value for all *F*_ST_ is less than 0.001.
(DOCX)

Table S3 **The diversity and genetic structure parameters (** ***F*** **-Statistics, global** ***R*** **-Statistics and heterozygosity values) corresponding to each of the six microsatellite loci.**
(DOCX)

Table S4 **The genetic parameters of populations of** ***D. cambodiana*** **in Hainan Island and Indochina Peninsular:** expected and observed heterozygosity (*H*_E_ and *H*_O_), and mean observed number of alleles per polymorphic locus (*A*), mean allelic richness per polymorphic locus (*A*_R_), number of private alleles (*A*_P_), *F*_IS_ and *F*_ST-SSR_ per population. *H*_E_ in * indicate significant deviations from HWE (at 0.05 significance level). The negative value of *F*_IS_ indicates heterozygosity excess.
(DOCX)

Table S5 **Analysis of population bottleneck based on IAM and TPM model of microsatellite evolution and Mode shift test for allele frequency distribution.** Significance of gene diversity excess (*H*e>*H*eq) was tested using Sign test and Wilcoxon signed ranks test (Luikart & Cornuet, 1998) based on 5000 replications. *P<0.05. NL = normal L-shaped distribution and MS = mode-shift in allele frequency distribution.
(DOCX)

File S1 **Supplementary materials and methods.**
(PDF)
