# Correction: Tertiary Origin and Pleistocene Diversification of Dragon Blood Tree (*Dracaena cambodiana*-Asparagaceae) Populations in the Asian Tropical Forests

**DOI:** 10.1371/annotation/e99f332e-5464-451a-9d46-e09a6bb1f239

**Published:** 2013-06-27

**Authors:** Jian-Li Zhao, Lu Zhang, Selvadurai Dayanandan, Shivaprakash Nagaraju, Dong-Mei Liu, Qiao-Ming Li

Figures 2, 3 and 4 were incorrectly switched. Figure 2 belongs with the title and legend appearing with Figure 3, Figure 3 belongs with the title and legend appearing with Figure 4, and Figure 4 belongs with the title and legend appearing with Figure 2. The titles and legends are in correct order. 

In addition, the affiliation of the third author was incorrect. Selvadurai Dayanandan is affiliated not with institution 3 but with institution 4, Department of Biology, Concordia University, Montreal, Canada. 

Finally, there was an error in Reference 11. The correct reference is: 11. An Z, Kutzbach J, Prell W, Porter S (2001) Evolution of Asian monsoons and phased uplift of the Himalaya-Tibetan Plateau since Late Miocene time. Nature 411: 62–66 

