# Supplementary figures and images for: Tertiary Origin and Pleistocene Diversification of Dragon Blood Tree (Dracaena cambodiana-Asparagaceae) Populations in the Asian Tropical Forests
Source: PLoS One. 2013 Apr 1;8(4):e60102. doi: 10.1371/journal.pone.0060102 (PMC3613351; doi:10.1371/journal.pone.0060102)

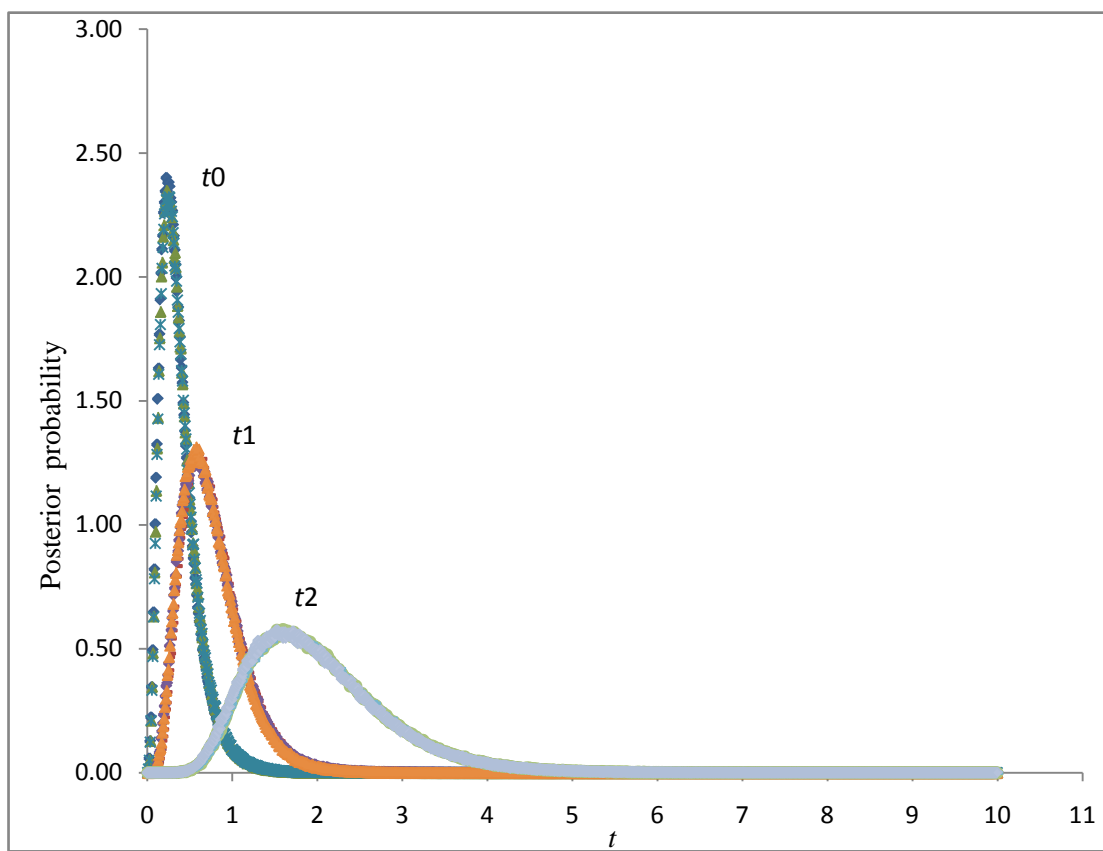

A

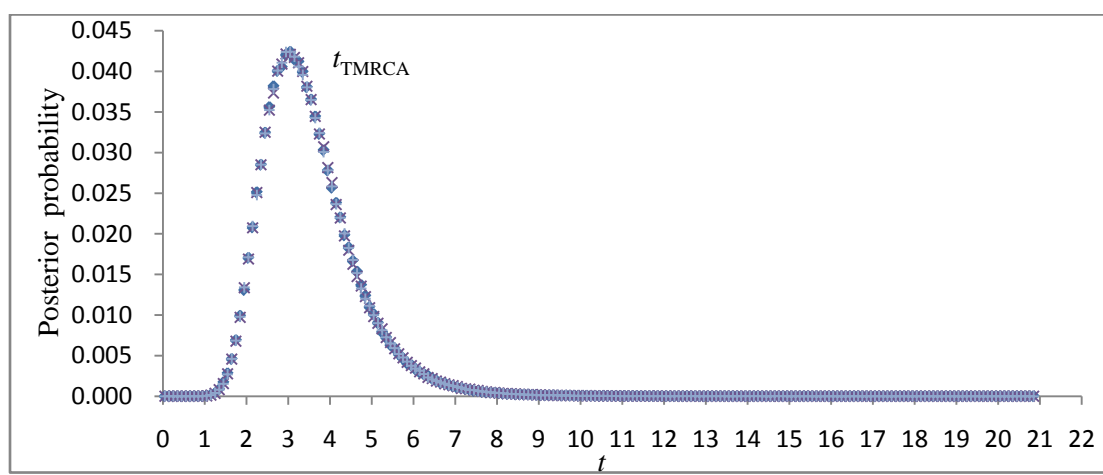

B

Supplement: Figure S1 — The posterior probability convergence of t 0, t 1, t 2 and t TMRCA. A: convergence of time parameters for each node in Figure 3. B: convergence of time parameter for the most recent common ancestor. (PDF) [file pone.0060102.s001.pdf]

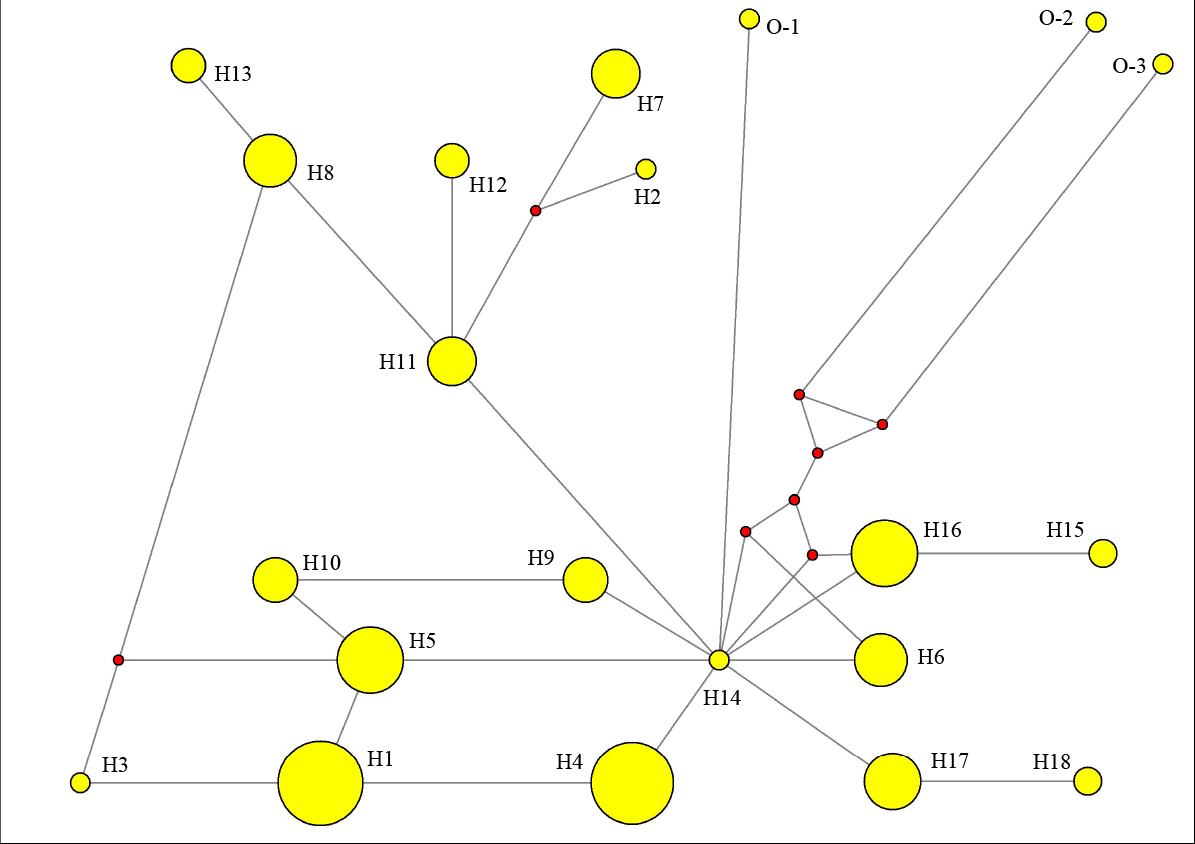

Supplement: Figure S2 — The phylogenetic network of haplotypes with outgroups. O-1 = Agava sp., O-2 = Yucca gloriosa, O-3 = Asparagus plumosus. Red dots are missing haplotypes or missing samples. (TIF) [file pone.0060102.s002.tif]

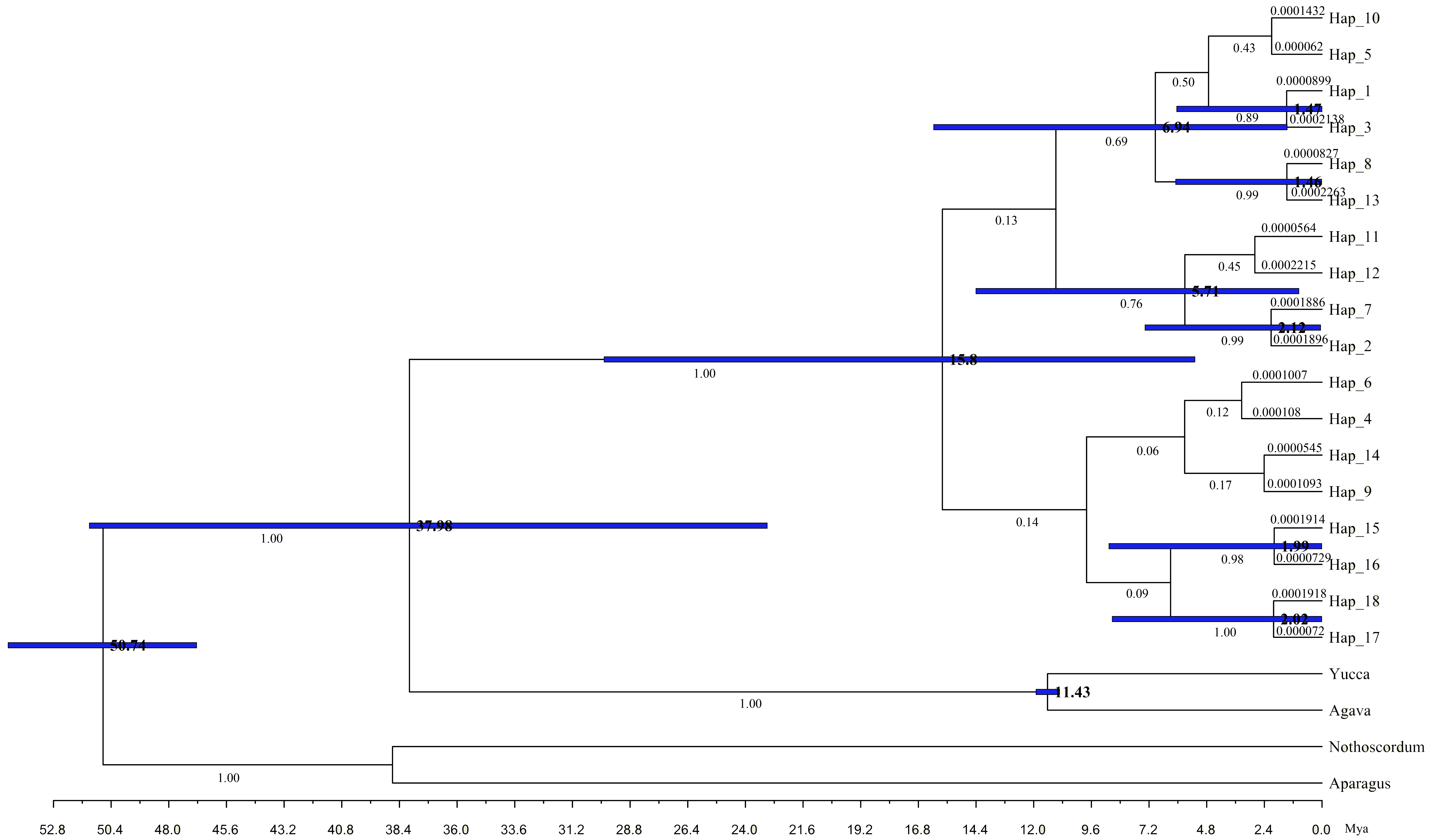

Supplement: Figure S3 — The bayesian tree for evolutionary rate estimation based on uncorrelated lognormal relaxed clock. Haps are the cpDNA haplotypes. The numbers above branches are the median rates of substitutions per site per million years. The numbers below the branches denotes the Bayesian posteriors. The height-median divergence time (boldfaces) and the 95%HPD time ranges (blue bars) marked at the nodes for the posteriors are more than 0.6. The coalescent times of the most recent common ancestor (T TMRCA) of 18 cpDNA haplotypes is 15.80 (5.29–29.88) Ma. (TIF) [file pone.0060102.s003.tif]
