# Supplementary material for: Tertiary Origin and Pleistocene Diversification of Dragon Blood Tree (Dracaena cambodiana-Asparagaceae) Populations in the Asian Tropical Forests
Source: PLoS One. 2013 Apr 1;8(4):e60102. doi: 10.1371/journal.pone.0060102 (PMC3613351; doi:10.1371/journal.pone.0060102)
Supplement: Table S1 — log10 Bayes factors of three different relaxed clock models. (DOCX) [file pone.0060102.s005.docx]

**Table S1** log10 Bayes factors of three different relaxed clock models.

| Trace | ln P | S.E. | Random | Unex | Unlog |
| --- | --- | --- | --- | --- | --- |
| Random | -4490.989 | +/- 0.204 | - | -1.172 | -1.521 |
| Unex | -4488.29 | +/- 0.212 | 1.172 | - | -0.349 |
| **Unlog** | **-4487.487** | **+/- 0.172** | **1.521** | **0.349** | **-** |
