# Supplementary material for: Tertiary Origin and Pleistocene Diversification of Dragon Blood Tree (Dracaena cambodiana-Asparagaceae) Populations in the Asian Tropical Forests
Source: PLoS One. 2013 Apr 1;8(4):e60102. doi: 10.1371/journal.pone.0060102 (PMC3613351; doi:10.1371/journal.pone.0060102)
Supplement: Table S2 — Pairwise genetic differentiation ( F ST) among four groups. The permutations for significance tests were 1023. P-value for all F ST is less than 0.001. (DOCX) [file pone.0060102.s006.docx]

**Table S2** Pairwise genetic differentiation (*F*_ST_) among four groups. The permutations for significance tests were 1023. *P*-value for all *F*_ST_ is less than 0.001.

| Group | S-I | NE-I | N-I | HN |
| --- | --- | --- | --- | --- |
| S-I | 0.00000 |  |  |  |
| NE-I | 0.22174 | 0.00000 |  |  |
| N-I | 0.29889 | 0.28875 | 0.00000 |  |
| HN | 0.31811 | 0.30890 | 0.37930 | 0.00000 |
