# Supplementary material for: Tertiary Origin and Pleistocene Diversification of Dragon Blood Tree (Dracaena cambodiana-Asparagaceae) Populations in the Asian Tropical Forests
Source: PLoS One. 2013 Apr 1;8(4):e60102. doi: 10.1371/journal.pone.0060102 (PMC3613351; doi:10.1371/journal.pone.0060102)
Supplement: Table S3 — The diversity and genetic structure parameters ( F -Statistics, global R -Statistics and heterozygosity values) corresponding to each of the six microsatellite loci. (DOCX) [file pone.0060102.s007.docx]

**Table S3** The diversity and genetic structure parameters (*F*-Statistics, global *R*-Statistics and heterozygosity values) corresponding to each of the six microsatellite loci.

| **Locus** | ***F*_IS_** | ***P*-value** | ***F*_SC_** | ***P*-value** | ***F*_CT_** | ***P*-value** | ***F*_IT_** | ***P*-value** | ***H*_O_** | ***H*_E_** | ***F*_ST_** | ***R*_ST_** |
| --- | --- | --- | --- | --- | --- | --- | --- | --- | --- | --- | --- | --- |
| Locus 03 | 0.090 | 0.038 | 0.328 | 0.000 | -0.025 | 0.972 | 0.366 | 0.000 | 0.618 | 0.940* | 0.450 | 0.875 |
| Locus 06 | -0.038 | 0.815 | 0.240 | 0.000 | 0.010 | 0.301 | 0.213 | 0.000 | 0.744 | 0.945* | 0.245 | 0.639 |
| Locus 140 | 0.152 | 0.000 | 0.187 | 0.000 | 0.042 | 0.019 | 0.341 | 0.000 | 0.628 | 0.960* | 0.216 | 0.272 |
| Locus 437 | -0.054 | 0.876 | 0.508 | 0.000 | 0.014 | 0.229 | 0.495 | 0.000 | 0.494 | 0.938* | 0.521 | 0.845 |
| Locus 460 | -0.127 | 1.000 | 0.368 | 0.000 | 0.011 | 0.068 | 0.304 | 0.000 | 0.676 | 0.955* | 0.406 | 0.954 |
| Locus 501 | 0.184 | 0.000 | 0.153 | 0.000 | -0.001 | 0.466 | 0.317 | 0.000 | 0.662 | 0.951* | 0.142 | 0.186 |
