# Supplementary material for: Tertiary Origin and Pleistocene Diversification of Dragon Blood Tree (Dracaena cambodiana-Asparagaceae) Populations in the Asian Tropical Forests
Source: PLoS One. 2013 Apr 1;8(4):e60102. doi: 10.1371/journal.pone.0060102 (PMC3613351; doi:10.1371/journal.pone.0060102)
Supplement: Table S4 — The genetic parameters of populations of D. cambodiana in Hainan Island and Indochina Peninsular: expected and observed heterozygosity (H E and H O), and mean observed number of alleles per polymorphic locus (A), mean allelic richness per polymorphic locus (A R), number of private alleles (A P), F IS and F ST-SSR per population. H E in * indicate significant deviations from HWE (at 0.05 significance level). The negative value of F IS indicates heterozygosity excess. (DOCX) [file pone.0060102.s008.docx]

**Table S4** The genetic parameters of populations of *D. cambodiana* in Hainan Island and Indochina Peninsular: expected and observed heterozygosity (*H*_E_ and *H*_O_), and mean observed number of alleles per polymorphic locus (*A*), mean allelic richness per polymorphic locus (*A*_R_), number of private alleles (*A*_P_), *F*_IS_ and *F*_ST-SSR_ per population. *H*_E_ in * indicate significant deviations from HWE (at 0.05 significance level). The negative value of *F*_IS_ indicates heterozygosity excess.

| Code | *H*_O_ | *H*_E_ | *A* | *A*_R_ | *A*_P_ | *F*_IS_ | *F*_ST-SSR_ |
| --- | --- | --- | --- | --- | --- | --- | --- |
| **S-I** | **0.58** | **0.631** | **6.111** | **5.072** | **15** | **0.08** | **0.343** |
| TL | 0.44 | 0.669* | 6.333 | 4.954 | 6 | 0.345 | 0.317 |
| BA | 0.47 | 0.644* | 6.5 | 5.289 | 4 | 0.273 | 0.314 |
| KP | 0.726 | 0.608 | 5.833 | 4.973 | 5 | -0.198 | 0.341 |
| **NE-I** | **0.709** | **0.717** | **7.889** | **6.135** | **46** | **0.011** | **0.298** |
| PX | 0.736 | 0.776 | 7.333 | 6.205 | 14 | 0.052 | 0.259 |
| JC | 0.565 | 0.553 | 4 | 3.29 | 8 | -0.023 | 0.368 |
| HF | 0.75 | 0.827 | 12.333 | 8.912 | 24 | 0.094 | 0.241 |
| **N-I** | **0.678** | **0.712** | **6.2** | **5.1** | **37** | **0.047** | **0.324** |
| JG | 0.587 | 0.697* | 5.833 | 4.828 | 5 | 0.161 | 0.298 |
| ML | 0.805 | 0.706* | 5.333 | 4.544 | 7 | -0.143 | 0.249 |
| NX | 0.661 | 0.750* | 7.5 | 6.03 | 10 | 0.121 | 0.272 |
| MM | 0.694 | 0.799* | 7.333 | 5.766 | 10 | 0.133 | 0.252 |
| LA | 0.522 | 0.640* | 5 | 4.334 | 5 | 0.187 | 0.327 |
| **HN** | **0.484** | **0.576** | **4.667** | **4.403** | **9** | **0.17** | **0.395** |
| DF | 0.444 | 0.544* | 5.333 | 4.249 | 4 | 0.187 | 0.376 |
| SY | 0.405 | 0.674* | 5.5 | 4.557 | 5 | 0.405 | 0.326 |
| **Over all**  **(Mean±SD)** | **0.637 (0.083)** | **0.948 (0.009)** | **36.667 (4.676)** |  | **107** | **0.050** | **0.329** |
