# Supplementary material for: Tertiary Origin and Pleistocene Diversification of Dragon Blood Tree (Dracaena cambodiana-Asparagaceae) Populations in the Asian Tropical Forests
Source: PLoS One. 2013 Apr 1;8(4):e60102. doi: 10.1371/journal.pone.0060102 (PMC3613351; doi:10.1371/journal.pone.0060102)
Supplement: Table S5 — Analysis of population bottleneck based on IAM and TPM model of microsatellite evolution and Mode shift test for allele frequency distribution. Significance of gene diversity excess (He>Heq) was tested using Sign test and Wilcoxon signed ranks test (Luikart & Cornuet, 1998) based on 5000 replications. *P<0.05. NL = normal L-shaped distribution and MS = mode-shift in allele frequency distribution. (DOCX) [file pone.0060102.s009.docx]

**Table S5** Analysis of population bottleneck based on IAM and TPM model of microsatellite evolution and Mode shift test for allele frequency distribution. Signiﬁcance of gene diversity excess (*H*e > *H*eq) was tested using Sign test and Wilcoxon signed ranks test (Luikart & Cornuet, 1998) based on 5000 replications. * *P* < 0.05. NL = normal L-shaped distribution and MS = mode-shift in allele frequency distribution.

| **Population Name** | **Sign test (two tailed p-value)** | | **Wilcoxon signed rank test (two tailed p-value)** | | **Allele frequency distribution** |
| --- | --- | --- | --- | --- | --- |
|  | **IAM** | **TPM** | **IAM** | **TPM** |  |
| **Southern Indochina (S-I)** | | | | | |
| Ubon, Thailand (TL) | 0.178 | 0.510 | 0.047 | 0.078 | NL |
| Batdambang, Cambodia (BA) | 0.075 | 0.328 | 0.030* | 0.094 | NL |
| Kampot, Cambodia (KP) | 0.271 | 0.280 | 0.063 | 0.094 | NL |
| **Northeastern of Indochina (NE-I)** | | | | | |
| Pingxiang, Guangxi Province, China (PX) | 0.044* | 0.232 | 0.016* | 0.031* | NL |
| Jingxi, Guangxi Province, China (JC) | 0.166 | 0.507 | 0.080 | 0.562 | NL |
| Haiphong, Vietnam (HF) | 0.049* | 0.180 | 0.016* | 0.078 | NL |
| **Northern Indochina (N-I)** | | | | | |
| Jinggu, Yunnan Province, China (JG) | 0.197 | 0.214 | 0.078 | 0.437 | NL |
| Menglian, Yunnan Province, China (ML) | 0.026* | 0.031* | 0.016* | 0.016* | MS |
| Zhenkang, Yunnan Province, China (NX) | 0.228 | 0.226 | 0.437 | 0.437 | NL |
| Mengyuan, Yunnan Province, China (MM) | 0.041* | 0.043* | 0.016* | 0.016* | MS |
| Louang Phrabang, Laos (LA) | 0.147 | 0.172 | 0.031* | 0.078 | MS |
| **Hainan Island (HN)** | | | | | |
| Dongfang, Hainan Province, China (DF) | 0.311 | 0.320 | 0.219 | 0.625 | NL |
| Sanya, Hainan Province, China (SY) | 0.028* | 0.177 | 0.016* | 0.031* | NL |
